# Pragmatic Return to Effective Dental Infection Control through Triage and Testing (PREDICT): A feasibility study to improve dental office safety

**DOI:** 10.21203/rs.3.rs-3011647/v1

**Published:** 2023-09-07

**Authors:** Janine Fredericks-Younger, Cecile Feldman, Veerasathpurush Allareddy, Ellen Funkhouser, MaryAnn McBurnie, Cyril Meyerowitz, Pat Ragusa, Julie Chapman-Greene, Modupe Coker, Daniel H Fine, Maria Laura Gennaro, Gayathri Subramanian

**Affiliations:** Rutgers School of Dental Medicine; Rutgers School of Dental Medicine; University of Illinois Chicago College of Dentistry; UA: The University of Alabama; Kaiser Permanente Center for Health Research; University of Rochester School of Medicine and Dentistry; University of Rochester School of Medicine and Dentistry; Rutgers School of Dental Medicine; Rutgers School of Dental Medicine; Rutgers School of Dental Medicine; Rutgers New Jersey Medical School; Rutgers School of Dental Medicine

**Keywords:** Dentistry, Infection Control, Infectious Disease, Dental Public Health, Risk Mitigation Occupational Safety, Patient Safety, Practice management, SARS-CoV-2, Point-of care testing

## Abstract

**Background:**

The COVID-19 pandemic highlights the need for practitioners to enhance workflows to increase safety and mitigate risk. As dental practice creates a highly aerosolized environment, pre-visit testing for SARS-CoV-2 has the potential to be an effective mitigation strategy to minimize disease transmission in dental offices. The Pragmatic Return to Effective Dental Infection Control through Testing (PREDICT) Feasibility Study examined the potential, logistics, and impact related to laboratory-based PCR viral testing and point-of-care (POC) antigen testing.

**Methods:**

Dental healthcare workers (DHCWs) and patients in four dental offices within the National Dental Practice-based Research Network participated in this prospective study. In addition to electronic surveys, participants in two offices completed POC testing, while participants in two offices used lab based PCR methods to detect SARS-CoV-2 infection. For this feasibility study, analysis was limited to descriptive measures. Median and interquartile ranges were reported for Likert scale responses and mean and standard deviation for continuous variables

**Results:**

Forty-one of forty-three consented patients and twenty-eight of twenty-nine DHCWs completed the protocol. Descriptive statistics calculations including median and interquartile ranges revealed (1) saliva, tongue epithelial cells and nasal swabs were the most desirable specimens for testing for groups (2) both LAB and POC protocols took similar amounts of total time to complete the full testing protocol and (3) DHCWs and patients reported feeling more comfortable when both groups were tested.

**Conclusions:**

This feasibility study suggests that pre-visit SARS-CoV-2 testing can be effectively implemented into dental practice workflows and positively impact perception of safety for DHCWs and patients, though a larger scale, network study is necessary for generalizability of results. As new virulent infectious diseases continue to emerge, preparing dental personnel to employ an entire toolbox of risk mitigation strategies, including testing, may have the potential to decrease dental practice closure time, maintaining continuity of dental care services for patients.

**Trial registration:**

This trial was registered on ClinicalTrials.gov: NCT05123742.

## Background

The emergence of the severe acute respiratory syndrome coronavirus 2 (SARS-CoV-2 virus) has reminded the world of the dangers humanity faces from novel infections.^(1, 2)^ First identified in December 2019^(3)^, the SARS-CoV-2 virus upended our day-to-day activities, initially driving many into isolation, causing interruptions in the provision of essential medical and dental care ^(4–10)^ As the disease shifts from pandemic to endemic^(11)^, SARS-CoV-2 continues to mutate.^(12, 13)^ While initially virulent with high mortality, the virus is less virulent though morbidity and mortality remains high.^(14, 15)^

Throughout history, the emergence of novel pathogens have changed practice patterns and will continue to challenge and alter current healthcare practice. For example, in the late 1980s, the human immunodeficiency virus significantly impacted personal protective equipment (PPE) standards.^(16, 17)^ More recently, the halting of routine dental care services at the start of the Coronavirus disease of 2019 (COVID-19) pandemic illustrated the need to have mechanisms in place to mitigate risk and ensure continued safety of dental healthcare workers (DHCWs) and patients.^(18–20)^ Infectious disease testing is one important measure that can potentially curtail interruptions in care. Several months after the start of the pandemic, polymerase chain reaction (PCR) and antigen point-of-care (POC) testing became available.^(21)^ Hospitals and medical offices quickly incorporated these testing technologies into their practice workflows to increase safety and minimize in-office disease transmission for staff and patients. ^(22–24)^ Dental practices, however, were slow to adopt this mitigation strategy^(25)^, despite the nature of dental procedures, many being aerosol generating using of high-speed handpieces and ultrasonic scalers, compounding the potential risk of SARS-CoV-2 transmission within the dental office.^(26–32)^

When a virus with high morbidity and/or mortality is widely circulating within a community, testing is a key mitigation strategy that should be considered within dental practice to help prevent in-office transmission.^(33–35)^ By identifying infected individuals and halting person-to-person contact through testing, both DCHWs and patients can have an increased perception of safety within the dental practice environment.^(33, 34)^

The Pragmatic Return to Effective Dental Infection Control through Triage and Testing (PREDICT) Feasibility study^(36)^ was designed to examine the feasibility for implementing two COVID-19 testing strategies [lab-based polymerase chain reaction (PCR) and point-of-care testing (POC)] in dental offices. PREDCIT sought to identify advantages and potential barriers for each testing method and evaluate the impact of each strategy on patients’ and DHCWs’ perceptions of safety in dental offices. Study aims included: (1) To determine DHCW and Patient willingness to participate, (2) To determine DHCW and Patient willingness/ability to follow thru with triage, testing and survey administration procedures and (3) To determine ease of use of electronic survey instruments for both the DHCWs and patient participants. Results of this feasibility study provided preliminary data to inform the development of a large network-wide study that seeks to identify key mitigation strategies to prevent SARS-CoV-2 or novel infectious agents that may affect safety in a dental office.

## Methods

The PREDICT Feasibility Study was conducted within the National Dental Practice-Based Research Network (PBRN). Funded by the National Institute of Dental and Craniofacial Research of the National Institutes of Health, the PBRN consists of over 7,000 dental professionals across the United States who collaborate and conduct practice-based research.^(37, 38)^ Within this rich and diverse network, members are committed to advancing knowledge of dental practice by pursuing pragmatic approaches to answer important clinical questions.^(39, 40)^ Practice-based research explores answers to questions in actual practice environments where patient and dental provider preferences and biases influence decisions and outcomes.

### Approach:

The PREDICT Feasibility Study required participation of DHCWs and patients within dental practices. Four clinician investigators in the National Dental PBRN were recruited to participate. Each of the investigators worked within a dental office/practice with at least five DHCWs, each of which had the option to engage in or decline participation. All interested DHCWs in each of the four offices were consented by a PBRN Research Coordinator. All DHCW and patient participants were selected based on the following inclusion criteria: over 18 years of age, able to understand English, and able to sign consent. Exclusion criteria included individuals who previously participated in a prior COVID-19 testing feasibility study.

### Interventions:

**Figure 1:**
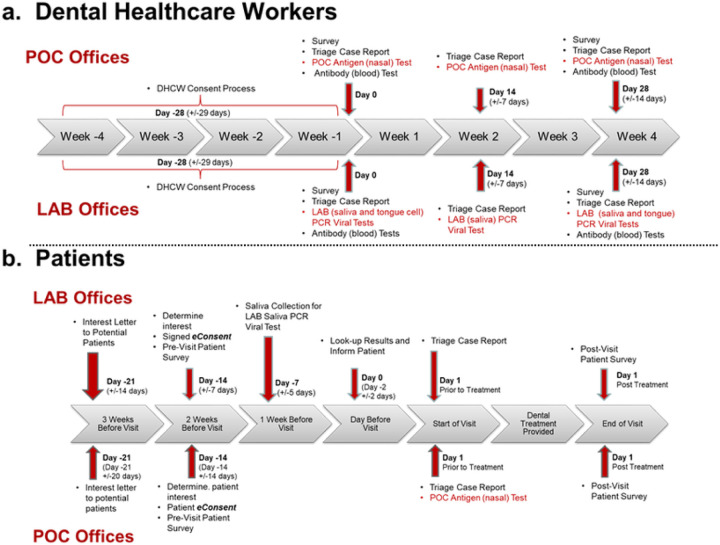
PREDICT Protocols: LAB and POC protocols for dental healthcare workers and the LAB and POC protocols for patient participants

There were two testing groups for DHCWs and patients: POC and LAB. Two offices were designated as LAB offices implementing the LAB protocol (PCR testing) for DHCWs and patients, while two offices were designated as POC offices testing the POC protocol. In total, four protocols were developed. Figure 1a illustrates the LAB and POC protocols for DHCWs and Fig. 1b illustrates the LAB and POC protocols for patients.

- DHCW – LAB protocol: DHCWs were consented by the PBRN Research Coordinator. On day 1 (start-of-study), DHCW participants completed a start-of-study survey, a symptom triage report and collected saliva, tongue epithelium and capillary blood samples which were sent to the lab for processing. Two weeks later, a second symptom triage report was completed and saliva and tongue specimens were collected. Finally, 2 weeks later, a third symptom triage report was completed, saliva, tongue epithelium cells and capillary blood sample collected along with completion of an end-of-study survey and study feasibly survey. SARS-CoV-2 test results along with antibody IgG and IgM results were made available to the PBRN investigator for sharing with dental office personnel as soon as available from the viral and antibody processing laboratories.
- DHCW – POC Protocol: DHCWs are consented by the PBRN Research Coordinator. On day 1 (start-of-study), DHCW participants completed a start-of-study survey, a symptom triage report, performed the POC SARS-CoV-2 antigen test, and provided capillary blood samples. Two weeks later, a second symptom triage report was completed and with the POC test repeated. Finally, 2 weeks later, a third symptom triage report was completed, POC test and capillary blood specimen collection repeated along with completion of an end-of-study survey and study feasibly survey.
- PATIENT-LAB Protocol: After written informed consent was obtained by a PBRN investigator, patients were asked to complete pre-visit questionnaire and were sent salvia collection kits. One week prior to their visit patients were requested to collect their saliva sample and drop the sample off at their dental office, which then forwarded the sample to the lab for analysis. Lab results were forwarded to the PBRN practitioner to inform patient participants prior to their dental visit. The symptom triage report was completed upon reporting for their dental visit. At the completion of their dental visit, a post-visit survey was completed along with a study feasibly questionnaire.
- PATIENT-POC Protocol: After written informed consent was performed by a PBRN investigator, patients were asked to complete a pre-visit questionnaire. Upon reporting for their visit, a symptom triage report was completed and the POC SARS-CoV-2 antigen test completed. At the completion of their dental visit, a post-visit survey was completed along with a study feasibly questionnaire.

The Abbott BinaxNOW test SARS-CoV-2 was utilized within POC offices. A nasal swab specimen was collected and inserted into the Abbott BinaxNOW Covid-19 antigen card to test for nucleocapsid protein antigen, which is used to determine SAR-CoV-2 infection. Conversely, in LAB offices, saliva and tongue samples were obtained and sent to the University lab for processing. Specifically, genetic material was extracted from the saliva and tongue epithelium samples via polymerase chain reaction (PCR) test to detect the presence of SARS-CoV-2 RNA, which can indicate present or past SARS-CoV-2 infection.

In addition to testing, questionnaires and surveys were administered to all participants electronically using Research Electronic Data Capture (REDCap), a secure web application, which supports research data collection and operations. For DHCWs, questionnaires at the start and end of the study assessed the impact of regular testing on the perception of safety at two-week intervals. Start of study questions included demographics, PPE used in the office, work practice controls used in the office, importance of triage and testing, importance of PPE measures, perceptions of safety and comfort in the workplace, safety culture in the office, SARS-CoV-2 testing preferences, dentist’s role in SARS-CoV-2 testing, and willingness to test in the office. The DHCW End of Study Survey included questions related to the importance of triage and testing, importance of PPE measures, perceptions of safety and comfort in the workplace, safety culture in the office, SARSCoV-2 testing preferences, dentist’s role in SARS-CoV-2 testing, willingness to test in the office, and vaccinations. The DHCW Participation Survey explored perceptions related to study participation including survey and testing logistics.

Similarly, patient pre- and post-visit patient questionnaires examined their beliefs and attitudes pre- and post-visit. The Patient Pre-visit Questionnaire investigated perceptions of safety and comfort, reasons for delaying dental care, concerns about returning to dental care, safety precautions valued, importance of triage and testing, and demographics. The Patient End-of-Visit Survey explored perceptions with testing preferences, PPE observed, environmental controls observed, concerns about returning to dental care, safety precautions valued, importance of triage and testing, likelihood of reporting symptoms, dentist’s role in COVID-19 testing and vaccinations. The Patient Participation Survey probed perceptions related to study participation including ease of survey and testing logistics.

### Outcome Measures and Statistical Analysis

Outcome measures included both process and “effect of intervention” impact measures. Process measures included time required to collect and process specimens, ability to complete the protocol within each window, length of time between obtaining the specimen and obtaining the results and ability to obtain SARS-CoV-2 viral and antigen results prior to start of the dental visit. Effect of intervention outcomes included sense of safety using the numeric rating scale (NRS), if 1) patients are tested, 2) DHCWs are tested and 3) both patients and DHCWs tested. Data related to specimen and test type preferences, willingness and amount to pay and required specificity and sensitivity levels were also collected as responses could significantly impact the design of testing protocols in dental offices.

For this feasibility study, analysis was limited to descriptive measures. Median and interquartile ranges were reported for Likert scale responses and mean and standard deviation for continuous variables. Separate results were reported for DHCWs and patients as protocols differed slightly for the groups (e.g., patients were queried before and after their visit and DHCWS were queried 3 times at 2-week intervals). Comparisons between LAB and POC DHCWs and patients were not performed as the number of participants was limited. Descriptive statistics were calculated using JMP Pro 16 and SAS.

### Ethical Considerations:

Human subject protection review was conducted and approved by the University Institutional Review Board. As PBRN investigators were dentists, testing was performed as screening for SARS-CoV-2, not to definitively diagnose COVID-19. Investigators were encouraged to refer positive patients or DHCWs to their primary care providers.

## Results

### Study Participants:

**Figure 2:**
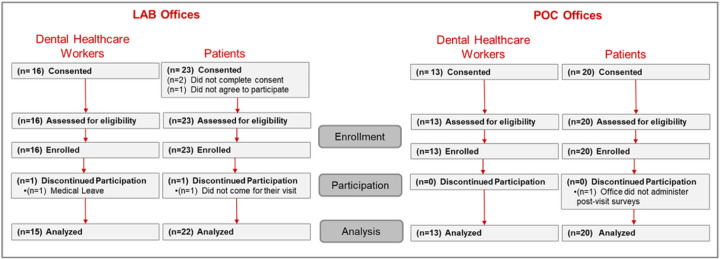
Participant Enrollment and Completion Status for dental healthcare worker and patient participants in the LAB and POC designated offices

Twenty-nine (29) DHCWs and 43 patients were consented (Figure 2), with 28 DCHWs and 41 patients completing the protocol. One DHCW was lost due to a non-COVID-19 related illness. One patient was consented but failed to report for their clinical visit, while another patient did not complete the post-visit surveys.

Participant demographics from the four pilot offices revealed majority of the DHCW participants were Caucasian (86%) and non-Hispanic (79%). Similarly, patient participants were predominantly Caucasian (88%) and non-Hispanic (84%). The majority of participants lived in suburban surroundings with most DHCWs and patients having a household income of more than $100,000. Mean age of DHCWs was 50 while the mean age of patients was 57. The twenty-nine DHCW participants had varied practice roles with the majority working chairside including twelve (41%) dental assistants, five (17%) owner dentists, four (14%) associate dentists, five (17%) dental hygienists. The majority of DHCW (90%) had some college education with twelve (41%) having participated in graduate level education. The majority of patient participants had engaged in college level coursework (30% completing some college, 35% Bachelor’s degree, and 21% with a graduate degree).

### COVID-19 History and Concern of Contracting COVID-19: (Table 1)

One fourth of DHCWs and patients reported a history of a positive COVID-19 diagnosis prior to participation. (Table 1). Patients reported more household members having been diagnosed with COVID-19 than DHCWs. Almost three quarters of DHCWs reported office mates having been diagnosed with COVID-19. Related to vaccinations, 86% of DHCWS and 93% of patients reported having received at least the first dose of a COVID-19 vaccination.

Related to perceptions of transmissibility within the dental office environment, 69% of DHCWs and 63% of patients indicated some degree of concern of contracting COVID-19 from patients in the dental office environment. Similarly, 69% of DHCWs and 49% of patients had some degree of concern of contracting COVID-19 from dental office personnel, with patients concerned to a lesser extent.

### COVID-19 Test Results:

Of the 41 patient participants, two tested positive for SARS-CoV-2 infection: one patient in the POC group (5.0%) and one in the LAB group (4.8%). For the 28 DHCWs who tested three times within a four week period, several DHCWs tested positive for SARS-CoV-2 infection. At both the start and end of the study, there was at least one DHCW identified as positive to SARS-CoV-2 through PCR (LAB). At the start of the study, two workers were identified as SARS-CoV-2 positive (12.5%) through PCR testing using both saliva and tongue specimens, though both workers were asymptomatic and reported having been diagnosed with COVID-19 several weeks prior. At the end of study, PCR testing results varied by specimen type as PCR testing using tongue epithelial cells identified three cases whereas PCR testing using saliva specimens did not for the same individual.

### COVID-19 Testing Preferences: (Table 2)

Both DHCWs and patients found venous blood was the least desirable specimen collection method for COVID-19 testing. Saliva, tongue epithelial cells and nasal swabs were rated the most desirable specimens for testing. DHCWs preferred POC testing in the dental office. Conversely, patients preferred collecting their specimen at home and mailing the specimen to a lab for processing with POC testing in the dental office was slightly less desirable. Dropping off collected specimens at the dental office and going to a commercial lab for specimen collection and laboratory processing was the least desirable method.

Overall, both participants reported feeling more comfortable being in an office with both DHCWs and patients being tested. Feeling of safety ratings decreased for both groups of participants when testing was limited to DHCWS and decreased even more significantly when testing was limited to patients only. When patients were asked if they preferred to go to an office where COVID-19 testing was regularly performed, 75% reported a preference to going to an office where patients and staff are tested. DHCWs were willing to require patients to pay a median rate of $18 for testing, while patients were willing to pay median rate of $15 to be tested. DHCWs were only willing to pay a median rate of $10 for their own testing.

When asked about minimum levels of sensitivity and specificity, DHCWs reported that tests needed to have a specificity and sensitivity of 85% or higher along with at false positives and false negatives being no greater than 50%.

### Screening Process Outcomes: (Table 3)

All screening processes, including specimen collection, and preparation and preparing specimens for shipping, and POC processing were considered easy to perform. Saliva specimen collection took longer than nasal POC specimen collection. Both the LAB and POC protocols took similar amounts of total time once all aspects of testing were included (specimen collection, drop off time for PCR testing, and processing time for POC testing).

## Discussion

This feasibility study suggests that dental offices can effectively implement SARS-CoV-2 testing into their workflows. Both dentists and patients reported feeling safer when both dental office personnel and patients were regularly tested. In addition, the majority of patient respondents prefer to go to an office where patients and staff are regularly tested. This evidence suggests that the implementation of SARS-CoV-2 testing can serve not only as an as effective mitigation strategy to decrease transmission but can positively impact the perception of safety for both DHCWs and patients within the office environment.

Results suggest that either testing method, Lab-based PCR or POC COVID-19 testing, can in fact, be built into dental practice workflows, though POC testing has multiple advantages. POC test kits are relatively inexpensive, within the range of willingness to pay, and provide results within 15 minutes. POC testing also has the added benefit of limiting the number of false positives as PCR testing picks up remnant viral particle presence for several months after a patient’s course of disease is over. While ease of use, low cost, and reliability make the POC testing favorable, practitioners who avail themselves to pre-visit POC testing should be cognizant, however, of some drawbacks. Limitations include testing administration logistics which require longer patient facing time (sample collection and analysis) and potential added operational costs (physical space and personnel required to enact workflows). The cost-benefit ratio of testing to potential missed appointments or work due to illness must be considered. Overall, in times of high prevalence with high morbidity and mortality, POC testing to mitigate risk of transmission may be an effective strategy to maintain a steady workforce, reduce office closures, and avoid disruption in dental care services.

There are several limitations to this study. As the purpose of this study was to develop the methodology for a large-scale PBRN based study, the sample size is small and results are not generalizable. There was limited diversity demographically, geographically and economically within the population tested. In addition, this feasibility study was conducted in an area of the US that is highly vaccinated and potentially more accepting of testing. Conclusions related to testing preference results are also limited as participants may not have been familiar nor had experience with all testing types. Another limitation specifically related to the logistics of PCR testing. While patients reported a preference for this protocol (specimen collection at-home and mail to lab), the management of this method for the dental office proved difficult. Continuity of care was hampered as results were not readily available. Lab processing efficiency impacted the receipt of timely results for patients with pending dental appointments. At the height of the pandemic, study laboratories engaged in PCR analysis had difficulty keeping up with demand and results were delayed. For dental providers who wish to implement PCR testing, perhaps a more viable method would be to require patients bear full responsibility for scheduling/completing testing at a commercial laboratory and reporting results to the dental office prior to treatment. Another alternative is to select the in-office POC testing method, which eliminates the need for outside processing and analysis.

## Conclusions

DHCWs and patients share concern about transmission of COVID-19 in the dental office and are receptive to SARS-CoV-2 testing as a mitigation strategy. It is feasible to implement SARS-CoV-2 testing in dental practice workflows. While the SARS-CoV-2 virus is now less virulent, SARS-CoV-2 point-of-care (POC) testing can be used as a model to investigate how dental practices can best prepare for the future. Future studies could contribute to the creation of standard testing practices for dental offices that can be adopted during times of high incidence of COVID-19, as well as, for the next novel virus.

## Figures and Tables

**Table 1:** COVID-19 History and Concern of Contracting COVID-19 for DHCW and Patient Participants

	DHCWs N = 29	Patients N = 43
COVID-19 History	N (%) Indicating Yes	N (%) Indicating Yes
Ever Been Diagnosed with COVID	7 (24%)	12 (28%)
Anyone you live with diagnosed with COVID	4 (14%)	14 (33%)
Anyone you work with diagnosed with COVID	21 (72%)	n/a
Vaccinated against COVID	25 (86%)	40 (93%)
Concern contracting COVID-19 from patients	N (%)	N (%)
Not at all	9 (31%)	16 (37%)
Mild	11 (38%)	16 (37%)
Moderate	7 (24%)	9 (21 %)
Severe	2 (7%)	2 (5%)
Concern contracting COVID-19 from Staff	N (%)	N (%)
Not at all	9 (31%)	22 (51%)
Mild	14 (48%)	10 (23%)
Moderate	5 (17%)	9 (21 %)
Severe	1 (3%)	2 (5%)

**Table 2:** SARS-CoV-2 Testing Preferences for DHCW and Patient Participants

TESTING PREFERENCE OUTCOMES		
	DHCWs N=28	Patients N=41
Specimen Preference (1-most to 6-least preferred)	Median (IQR)	Median (IQR)
Saliva	1 (1 – 2)	2 (1 – 3)
Tongue Epithelial Cells	2 (1 – 3)	2 (1 – 3)
Nasal Swab	2.5 (2 – 3)	2 (1 – 3)
Nasal Pharyngeal Swab	4 (4 – 4)	4 (3 – 5)
Finger Stick	5 (5 – 5)	5 (4 – 5)
Venous	6 (6 – 6)	6 (6 – 6)
Testing Protocol Preference (1-most to 4-least preferred)	Median (IQR)	Median (IQR)
Home Test and Mail to Lab	2 (1 – 2.5)	1 (1 – 2)
POC in Office	1.5 (1 – 3)	2 (1 – 3)
Specimen to Dental Office and then to Lab	2 (2 – 3)	3 (2 – 3)
Specimen to Commercial Lab	4 (3.5 – 4)	4 (4 – 4)
How safe do you think you would feel if….	Median (IQR)	Median (IQR)
All DHCW regularly test AND all Patients tested prior to dental visit	85.5 (67 – 100)	97 (88 – 100)
Just DHWS are regularly tested	73.5 (50 – 88.5)	79 (65 – 97)
Just Patients are regularly tested	54.5 (50 – 85)	55 (37 – 78)
Cost Willing to Pay for COVID Test	Median (IQR)	Median (IQR)
DHCW Testing	$10 ($0 - $20)	n/a
Patient Testing	$18 ($10 - $36)	$15 ($9 - $25)
Desired Test Specificity and Sensitivity (scale 0 to 100)	Median (IQR)	
Lowest Acceptable Sensitivity (limited to DMD/DDSs) N = 9	90 (89 – 98)	n/a
Lowest Acceptable Specificity (limited to DMD/DDSs) N = 9	90 (89 – 95)	n/a
If you had a choice which office would you prefer to go to:		N (%)
Where patients and staff are tested	n/a	31 (76%)
Doesn’t make a difference if patients and staff are tested	n/a	10 (24%)
Patients and staff are NOT tested	n/a	0 (0%)

TESTING PROCESS OUTCOMES				
	PCR	POC	PCR	POC
Ease of Performance	n=15 N (%) Indicating Easy	n=12 N (%) Indicating Easy	n=22 N (%) Indicating Easy	n=19 N (%) Indicating Easy
Collection of Nasal Specimen	n/a	10 (83%)	n/a	19 (100%)
Processing of Nasal Specimen	n/a	7 (58%)	n/a	n/a
Collection of Saliva Specimen	14 (93%)	n/a	21 (95%)	n/a
Preparing and Packaging for Shipment Saliva Specimen	14 (93%)	n/a	n/a	n/a
Collection of Tongue Specimen	14 (93%)	n/a	n/a	n/a
Preparing and Packing for Shipping Tongue Specimen	13 (87%)	n/a	n/a	n/a

**Table 3:** Time Required for Testing by Test Type

Time Required	Patients			
	PCR (n=22)		POC (n=19)	
	Collect Saliva	Drop-Off Saliva	Nasal Swab	POC Results
5 Minutes	9 (41 %)	9 (43%)	19 (100%)	5 (26%)
10 Minutes	10 (45%)	7 (33%)	0 (0%)	0 (0%)
15 Minutes	2 (9%)	5 (24%)	0 (0%)	15 (74%)
20 Minutes	1 (5%)	0 (0%)	0 (0%)	0 (0%)
25 Minutes	0 (0%)	0 (0%)	0 (0%)	0 (0%)
30 Minutes	0 (0%)	0 (0%)	0 (0%)	0 (0%)
>30 Minutes	0 (0%)	0 (0%)	0 (0%)	0 (0%)

## Data Availability

The datasets generated and/or analyzed during the current study are maintained and made available by the National Dental PBRN [https://www.nationaldentalpbrn.org/resource-sharing/].

## Key messages regarding feasibility

- What uncertainties existed regarding feasibility?
  It was unknown whether infectious disease testing could be effectively implemented within dental practice workflows for both dental healthcare workers (DHCWs) and patients. Furthermore, it was unknown whether DHCWs and/or patients would be amenable to infectious disease testing as an effort to mitigate risk and what the impact of testing would be on perception of safety within the dental office.
- What are the key feasibility findings?
  Dental offices could effectively implement infectious disease testing in the dental practice workflows for both staff and patients. Both DHCWs and patients were amenable to both lab-based PCR and in-office point-of-care testing (POC) and reported feeling more comfortable being in an office in which both DHCWs and patients were tested. Patients would be willing to pay additional fees for testing.
- What are the implications of the feasibility findings for the design of the main study?
  In designing the main study, we aim to identify best practices for implementing infectious disease testing as a high impact risk mitigation strategy, which can be employed in future pandemics. We believe this can maximize the safety of DHCWs and patients from novel infections, while reducing office closures and workforce exodus. Results of the feasibility study suggests infectious disease testing can be adopted into practice workflows and has a positive impact on perception of safety; however, results may not be generalizable due to the homogeneity of the study participants as the feasibility study was conducted within only four PBRN offices in the same region. A larger, network study utilizing the six regions of the National Dental Practice-based Research Network would produce more generalizable results as demographics would reflect more geographic, socio-economic, racial, and ethnic diversity.

## Abbreviations

COVID-19: Coronavirus disease of 2019
DHCW: Dental Healthcare worker
NRS: Numeric rating scale
PBRN: Practice-based Research Network
PCR: Polymerase chain reaction
POC: Point-of-care
PPE: Personal protective equipment
PREDICT: Pragmatic Return to Effective Dental Infection Control through Triage and Testing study
SARS-CoV-2: Severe acute respiratory syndrome coronavirus 2

## References

[1] Petrovan SO, Aldridge DC, Bartlett H, Bladon AJ, Booth H, Broad S, Broom DM, Burgess ND, Cleaveland S, Cunningham AA, Ferri M, Hinsley A, Hua F, Hughes AC, Jones K, Kelly M, Mayes G, Radakovic M, Ugwu CA, Uddin N, Veríssimo D, Walzer C, White TB, Wood JL, Sutherland WJ. Post COVID-19: a solution scan of options for preventing future zoonotic epidemics. Biol Rev Camb Philos Soc. 2021 Dec;96(6):2694–715. 10.1111/brv.12774. Epub 2021 Jul 7. 34231315 PMC8444924

[2] Khan G. A novel coronavirus capable of lethal human infections: an emerging picture. Virol J. 2013 Feb;28:10:66. 10.1186/1743-422X-10-66. PMC3599982 23445530

[3] Telenti A, Hodcroft EB, Robertson DL. The Evolution and Biology of SARS-CoV-2 Variants. Cold Spring Harb Perspect Med. 2022 May 27;12(5):a041390. doi: 10.1101/cshperspect.a041390. 35444005 PMC9159258

[4] McAlister FA, Parikh H, Lee DS, Wijeysundera HC. Health Care Implications of the COVID-19 Pandemic for the Cardiovascular Practitioner. Can J Cardiol. 2022 Dec 5:S0828–282X (22)01051–0. doi: 10.1016/j.cjca.2022.11.014. Epub ahead of print. PMC9721374 36481398

[5] Cha AE, Cohen RA. Dental Care Utilization Among Adults Aged 18–64: United States, 2019 and 2020. NCHS Data Brief. 2022 Apr;(435):1–8. 35575758

[6] Scully AC, Joshi AP, Rector JM, Eckert GJ. Willingness and ability of oral health care workers to work during the COVID-19 pandemic. J Am Dent Assoc. 2021 Oct;152(10):791–9. 10.1016/j.adaj.2021.04.021. Epub 2021 May 4. 34344507 PMC8096172

[7] Kranz AM, Chen A, Gahlon G, Stein BD. 2020 trends in dental office visits during the COVID-19 pandemic. J Am Dent Assoc. 2021 Jul;152(7):535–541e1. 10.1016/j.adaj.2021.02.016. Epub 2021 Mar 9. 34023093 PMC7942140

[8] El-Boghdadly K, Cook TM, Goodacre T, Kua J, Blake L, Denmark S, McNally S, Mercer N, Moonesinghe SR, Summerton DJ. SARS-CoV-2 infection, COVID-19 and timing of elective surgery: A multidisciplinary consensus statement on behalf of the Association of Anaesthetists, the Centre for Peri-operative Care, the Federation of Surgical Specialty Associations, the Royal College of Anaesthetists and the Royal College of Surgeons of England. Anaesth 2021 Jul;76(7):940–6. doi: 10.1111/anae.15464. Epub 2021 Mar 18. PMC8250763 33735942

[9] Understaffed. and ready to hire, dentists face applicant shortages as they emerge from covid-19 pandemic [Internet]. [cited 2023 May 31]. Available from: https://adanews.ada.org/ada-news/2021/june/dentists-face-applicant-shortages-as-they-emerge-from-covid-19-pandemic.

[10] Kranz AM, Gahlon G, Dick AW, Stein BD. Characteristics of US Adults Delaying Dental Care Due to the COVID-19 Pandemic. JDR Clin Trans Res 2021 Jan;6(1):8–14. doi: 10.1177/2380084420962778. Epub 2020 Sep 27. 32985322 PMC7527908

[11] Biancolella M, Colona VL, Mehrian-Shai R, Watt JL, Luzzatto L, Novelli G, Reichardt JKV. COVID-19 2022 update: transition of the pandemic to the endemic phase. Hum Genomics. 2022 Jun 1;16(1):19. doi: 10.1186/s40246-022-00392-1. 35650595 PMC9156835

[12] Araf Y, Akter F, Tang YD, Fatemi R, Parvez MSA, Zheng C, Hossain MG. Omicron variant of SARS-CoV-2: Genomics, transmissibility, and responses to current COVID-19 vaccines. J Med Virol 2022 May;94(5):1825–32. doi: 10.1002/jmv.27588. Epub 2022 Jan 23. 35023191 PMC9015557

[13] Hadj Hassine I. Covid-19 vaccines and variants of concern: A review. Rev Med Virol. 2022 Jul;32(4):e2313. 10.1002/rmv.2313. Epub 2021 Nov 9. 34755408 PMC8646685

[14] Zhang JJ, Dong X, Liu GH, Gao YD. Risk and Protective Factors for COVID-19 Morbidity, Severity, and Mortality. Clin Rev Allergy Immunol. 2023 Feb;64(1):90–107. doi: 10.1007/s12016-022-08921-5. Epub 2022 Jan 19. 35044620 PMC8767775

[15] El-Shabasy RM, Nayel MA, Taher MM, Abdelmonem R, Shoueir KR, Kenawy ER. Three waves changes, new variant strains, and vaccination effect against COVID-19 pandemic. Int J Biol Macromol 2022 Apr 15;204:161–8. doi: 10.1016/j.ijbiomac.2022.01.118. Epub 2022 Jan 22. 35074332 PMC8782737

[16] Brondani M, Donnelly L. The HIV and SARS-CoV-2 Parallel in Dentistry from the Perspectives of the Oral Health Care Team. JDR Clin Trans Res 2021 Jan;6(1):40–6. doi: 10.1177/2380084420961089. Epub 2020 Sep 18. 32942933 PMC7502681

[17] National Research Council (US) Panel on Monitoring the Social Impact of the AIDS Epidemic. The Social Impact Of AIDS. In: Jonsen AR, Stryker J, editors. The United States. Washington (DC): National Academies Press (US); 1993. p. 25121219. 25121219

[18] Kaur H, Gupta H, Dadlani H, Kochhar GK, Singh G, Bhasin R, Kochhar AS, Alam MK. Delaying Intraoral Radiographs during the COVID-19 Pandemic: A Conundrum. Biomed Res Int 2022 Jan 12;2022:8432856. doi: 10.1155/2022/8432856. 35036440 PMC8753251

[19] Ilhan B, Bayrakdar IS, Baydar O, Guneri P. Is It Time to Consider Implementation of Telemedicine in Current Oral Health Care Services? Disaster Med Public Health Prep. 2022 Apr;16(2):423–4. doi: 10.1017/dmp.2020.503. Epub 2021 Mar 26. 33769240

[20] Chigurupati R, Panchal N, Henry AM, Batal H, Sethi A, D’innocenzo R, Mehra P, Krishnan DG, Roser SM. Considerations for Oral and Maxillofacial Surgeons in COVID-19 Era: Can We Sustain the Solutions to Keep Our Patients and Healthcare Personnel Safe? J Oral Maxillofac Surg. 2020 Aug;78(8):1241–56. doi: 10.1016/j.joms.2020.05.027. Epub 2020 May 24. 32479811 PMC7246053

[21] Lai CKC, Lam W. Laboratory testing for the diagnosis of COVID-19. Biochem Biophys Res Commun 2021 Jan 29;538:226–30. doi: 10.1016/j.bbrc.2020.10.069. Epub 2020 Oct 28. 33139015 PMC7598306

[22] He M, Peaper DR, Murray T, Ciaburri R, Doyle J, Loyal J. Implementation of Pre-Admission Caregiver Testing for COVID-19. Hosp Pediatr. 2022 Oct 1;12(10):e326–e329. doi: 10.1542/hpeds.2022-006715. 36047308

[23] Kidambi TD, Idos GE, Lin JL, Pre-Procedural COVID, Testing. The “New Normal”. Gastroenterology. 2021 May;160(6):2189–2190. doi: 10.1053/j.gastro.2020.06.085. Epub 2020 Jul 1. 32621902 PMC7328632

[24] Bence CM, Jarzembowski JA, Belter L, Berens RJ, Henrickson KJ, Hoffman GM, Jackson F, Kehl KS, Oldham KT, Scott JP, Tassone JC, Woger N, Yale E, Gourlay DM. COVID-19 pre-procedural testing strategy and early outcomes at a large tertiary care children’s hospital. Pediatr Surg Int 2021 Jul;37(7):871–80. doi: 10.1007/s00383-021-04878-2. Epub 2021 Mar 14. 33715083 PMC7955904

[25] Zellmer S, Bachmann E, Muzalyova A, Ebigbo A, Kahn M, Traidl-Hoffmann C, Frankenberger R, Eckstein FM, Ziebart T, Meisgeier A, Messmann H, Römmele C, Schlittenbauer T. One Year of the COVID-19 Pandemic in Dental Medical Facilities in Germany: A Questionnaire-Based Analysis. Int J Environ Res Public Health. 2021 Dec 24;19(1):175. doi: 10.3390/ijerph19010175. 35010434 PMC8750787

[26] Gherlone E, Polizzi E, Tetè G, Capparè P. Dentistry and Covid-19 pandemic: operative indications post-lockdown. New Microbiol. 2021 Jan;44(1):1–11. Epub 2020 Oct 31. 33135082

[27] Derruau S, Bouchet J, Nassif A, Baudet A, Yasukawa K, Lorimier S, Prêcheur I, Bloch-Zupan A, Pellat B, Chardin H, Jung S, On Behalf Of Task Force Covid-Collège National des EnseignantS En Biologie Orale Cnesbo-France. COVID-19 and Dentistry in 72 Questions: An Overview of the Literature. J Clin Med. 2021 Feb 16;10(4):779. doi: 10.3390/jcm10040779. 33669185 PMC7919689

[28] Butt RT, Janjua OS, Qureshi SM, Shaikh MS, Guerrero-Gironés J, Rodríguez-Lozano FJ, Zafar MS. Dental Healthcare Amid the COVID-19 Pandemic. Int J Environ Res Public Health 2021 Oct 20;18(21):11008. doi: 10.3390/ijerph182111008. 34769526 PMC8583530

[29] Patel B, Eskander MA, Ruparel NB. To Drill or Not to Drill: Management of Endodontic Emergencies and In-Process Patients during the COVID-19 Pandemic. J Endod. 2020 Nov;46(11):1559–69. 10.1016/j.joen.2020.08.008. Epub 2020 Aug 22. 32841654 PMC7443083

[30] Pan Y, Liu H, Chu C, Li X, Liu S, Lu S. Transmission routes of SARS-CoV-2 and protective measures in dental clinics during the COVID-19 pandemic. Am J Dent. 2020 Jun;33(3):129–34. 32470237

[31] Ge ZY, Yang LM, Xia JJ, Fu XH, Zhang YZ. Possible aerosol transmission of COVID-19 and special precautions in dentistry. J Zhejiang Univ Sci B. 2020 May;21(5):361–8. 10.1631/jzus.B2010010. Epub 2020 Mar 16. 32425001 PMC7089481

[32] Abramovitz I, Palmon A, Levy D, Karabucak B, Kot-Limon N, Shay B, Kolokythas A, Almoznino G. Dental care during the coronavirus disease 2019 (COVID-19) outbreak: operatory considerations and clinical aspects. Quintessence Int. 2020;51(5):418–429. doi: 10.3290/j.qi.a44392. 32328595

[33] Feldman CA, Fredericks-Younger J, Subramanian G, Gennaro ML, Coker MO, Fine DH. Severe acute respiratory syndrome coronavirus 2 screening to augment dental office and patient safety. J Am Dent Assoc. 2022 May;153(5):399–402. 10.1016/j.adaj.2021.12.011. Epub 2022 Jan 24. 35339265 PMC8784650

[34] Shirazi S, Stanford CM, Cooper LF. Testing for COVID-19 in dental offices: Mechanism of action, application, and interpretation of laboratory and point-of-care screening tests. J Am Dent Assoc. 2021 Jul;152(7):514–525.e8. doi: 10.1016/j.adaj.2021.04.019. Epub 2021 May 4. Erratum in: J Am Dent Assoc. 2021 Sep;152(9):719. 34176567 PMC8096195

[35] Lieberthal B, McCauley LK, Feldman CA, Fine DH. COVID-19 and Dentistry: Biological Considerations, Testing Strategies, Issues, and Regulations. Compend Contin Educ Dent. 2021 Jun;42(6):290–6. quiz 297. 34077663

[36] Fredericks-Younger J, Fine DH, Subramanian G, Coker MO, Meyerowitz C, Ragusa P, Allareddy V, McBurnie MA, Funkhouser E, Gennaro ML, Feldman CA. The Pragmatic Return to Effective Dental Infection Control Through Triage and Testing (PREDICT) Study: Protocol for a Prospective Clinical Study in the National Dental Practice-Based Research Network. JMIR Res Protoc. 2022 Aug 31;11(8):e38386. doi: 10.2196/38386. 35944181 PMC9439378

[37] The National Dental Practice-based Research Network. : Clinical Dental Studies [Internet]. 2023 [cited 2023 May 31]. Available from: https://www.nationaldentalpbrn.org/.

[38] Gordan VV, Makhija SK, Rindal DB, Meyerowitz C, Fellows JL, Ziegenfuss JY, Cochran DL, Hudak S, Gilbert GH, National Dental PBRN Collaborative Group. Leadership in practice-based research: The National Dental PBRN. J Dent. 2019 Aug;87:24–7. 10.1016/j.jdent.2019.05.009. Epub 2019 May 7. 31075374 PMC7020881

[39] Mungia R, Funkhouser E, Buchberg Trejo MK, Cohen R, Reyes SC, Cochran DL, Makhija SK, Meyerowitz C, Rindal BD, Gordan VV, Fellows JL, McCargar JD, McMahon PA, Gilbert GH, National Dental PBRN Collaborative Group. ;. Practitioner Participation in National Dental Practice-based Research Network (PBRN) Studies: 12-Year Results. J Am Board Fam Med. 2018 Nov-Dec;31(6):844–56. doi: 10.3122/jabfm.2018.06.180019. 30413541 PMC6936735

[40] Mjör IA. Controlled clinical trials and practice-based research in dentistry. J Dent Res. 2008 Jul;87(7):605. doi: 10.1177/154405910808700702. 18573977

